# Epithelial and Mesenchymal Features of Pancreatic Ductal Adenocarcinoma Cell Lines in Two- and Three-Dimensional Cultures

**DOI:** 10.3390/jpm12050746

**Published:** 2022-05-04

**Authors:** Yuuki Shichi, Fujiya Gomi, Norihiko Sasaki, Keisuke Nonaka, Tomio Arai, Toshiyuki Ishiwata

**Affiliations:** 1 Division of Aging and Carcinogenesis, Research Team for Geriatric Pathology, Tokyo Metropolitan Institute of Gerontology, Tokyo 173-0015, Japan; y_shichi@tmig.or.jp (Y.S.); gomif@tmig.or.jp (F.G.); nona_kei@tmig.or.jp (K.N.); 2 Research Team for Geriatric Medicine (Vascular Medicine), Tokyo Metropolitan Institute of Gerontology, Tokyo 173-0015, Japan; sasanori@tmig.or.jp; 3 Department of Pathology, Tokyo Metropolitan Hospital and Institute of Gerontology, Tokyo 173-0015, Japan; arai@tmig.or.jp

**Keywords:** pancreatic cancer, pancreatic ductal adenocarcinoma, cell line, two-dimensional culture, three-dimensional culture

## Abstract

Pancreatic ductal adenocarcinoma (PDAC) is an intractable cancer that is difficult to diagnose early, and there is no cure other than surgery. PDAC is classified as an adenocarcinoma that has limited effective anticancer drug and molecular-targeted therapies compared to adenocarcinoma found in other organs. A large number of cancer cell lines have been established from patients with PDAC that have different genetic abnormalities, including four driver genes; however, little is known about the differences in biological behaviors among these cell lines. Recent studies have shown that PDAC cell lines can be divided into epithelial and mesenchymal cell lines. In 3D cultures, morphological and functional differences between epithelial and mesenchymal PDAC cell lines were observed as well as the drug effects of different anticancer drugs. These effects included gemcitabine causing an increased growth inhibition of epithelial PDAC cells, while nab-paclitaxel caused greater mesenchymal PDAC cell inhibition. Thus, examining the characteristics of epithelial or mesenchymal PDAC cells with stromal cells using a 3D co-culture may lead to the development of new anticancer drugs.

## 1. Introduction

Pancreatic cancer is a malignant tumor disease with poor prognosis and a 5-year overall survival rate of approximately 11% [1]. The 5-year survival rate for pancreatic cancer in the 1990s was 3–4% [2]; therefore, even after a quarter of a century, it has only improved by a few percent. It is estimated that approximately 42,000 people in Japan and 62,000 people in the United States suffer from pancreatic cancer annually; in 2020, 466,000 people worldwide died from pancreatic cancer [3]. Currently, pancreatic cancer is the fourth leading cause of cancer-related deaths after lung, colorectal, and gastric cancer in Japan, and it is the third leading cause of cancer-related deaths after lung and colon cancer in the United States. By 2030, pancreatic cancer is projected to be the second leading cause of cancer-related deaths in the United States [4]. Age is a major risk factor for pancreatic cancer, with most patients diagnosed in their 70s and 80s [5]. With the rapidly aging population in developed countries, an increase in global pancreatic cancer-related deaths is expected.

Among all pancreatic malignancies, pancreatic ductal adenocarcinoma (PDAC) is the most common subtype, has high proliferative and metastatic potential in other organs, and accounts for approximately 90% of all pancreatic malignancies. Thus, pancreatic cancer and PDAC are often used interchangeably [6]. Although surgery is the only cure for PDAC, early diagnosis is difficult, with 80% of patients with PDAC diagnosed with inoperable tumors. Tumor biomarkers in the blood do not show high levels in most PDAC cases, and there are no diagnostic imaging methods that can easily observe the entire pancreas to detect early-stage cancer. PDAC is histologically classified as an adenocarcinoma, similar to most breast, colorectal, and prostate cancers. However, anticancer drugs and molecular-targeted therapies that are effective against these cancers are not effective against PDAC. Patients with PDAC who have undergone surgery have frequent recurrences and metastases after surgery, and the 5-year survival rate of postoperative patients is 15–20% [7]. Furthermore, there is no effective treatment for PDAC recurrence or metastasis.

Basic research on PDAC has mainly been conducted using cultured PDAC cell lines, experimental animals, and human surgical tissue specimens. Cultured PDAC cell lines were established from patients that had different gene mutations and expression profiles [8,9]. PDAC is usually cultured two-dimensionally (2D), and its morphology and function have been analyzed in this manner. However, PDAC cells proliferate three-dimensionally (3D) in both primary and metastatic lesions in the human body. Understanding the characteristics of 3D-cultured PDAC cell lines established from different patients is important for the development of basic research on the diagnosis and treatment of PDAC. In this review, we discuss the morphological and functional differences between PDAC cell lines cultured in 2D and 3D conditions. In 3D culture, the difference between individual PDAC cell lines was clearer than in 2D culture; in particular, the difference between epithelial and mesenchymal features of PDAC was discovered.

## 2. Overview of Human Pancreatic Cancer Cell Lines

Cell lines are ubiquitous experimental tools in cancer research. Cancer cell lines are developed from a patient’s cancer cells, and these lines are considered to meet the definition of tumors that grow autonomously and indefinitely. Based on these properties, cancer stem cells (CSCs) that have the capacity to self-renew are present in cultured cancer cell lines. Many cell lines have been used for pancreatic cancer research. The most frequently altered driver genes in PDAC include the oncogene *KRAS* and the three tumor suppressor genes *TP53*, *CDKN2A*, and *SMAD4*, which have been thoroughly characterized in human tissue samples [10,11,12,13]. It has been reported that TGF-β treatment induces lethal epithelial-mesenchymal transition (EMT) and tumor suppression through snail in PDAC cells with functional SMAD4. In PDAC cells with non-functional SMAD4, TGF-β-induced EMT through snail does not occur; instead, Sox4 and Klf5 are collectively involved in tumorigenesis [14]. Mutations in these four driver gene accumulate and progress from PanIN-1 (low-grade PanIN) to PanIN-3 (high-grade PanIN) in precancerous lesions and can lead to the development of pancreatic cancer [15]. Some PDAC cell lines have genetic abnormalities in all four driver genes, but other PDAC cell lines have different patterns of genetic alteration among the four driver genes [16]. Since PDAC cell lines are established from different patients, this suggests that the carcinogenesis process of PDAC is diverse. However, there are challenges in using cultured cell lines. PDAC cell lines were developed from unique cancer cells selected at the time of cell establishment and may not represent the characteristics observed in all patients with cancer. Moreover, culturing 3D pancreatic cancer cells on one plane is considered different from in vivo conditions [17]. Studies on PDAC cell lines also ignore inflammatory cells, fibroblasts, and fibrosis that are proximal to cancer cells in patients with PDAC.

**Figure 1:**
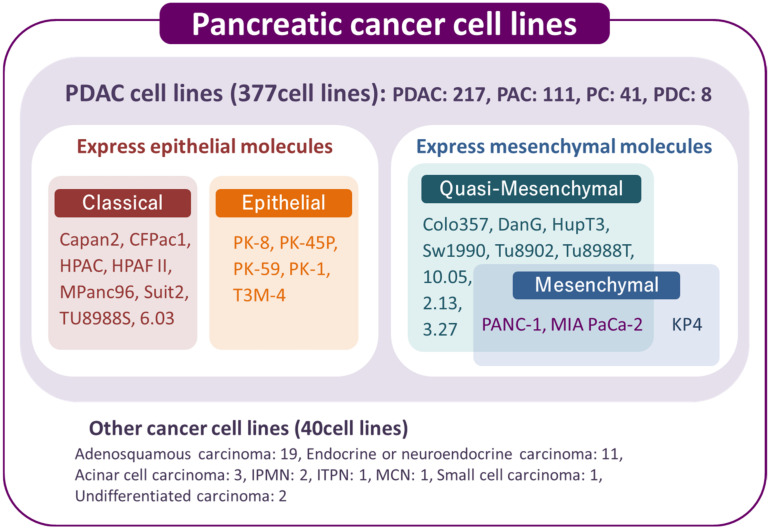
Pancreatic cancer cell lines listed in the Cellosaurus.

Several public cell banks supply cancer cell lines worldwide, such as the American Type Culture Collection (ATCC), European Collection of Cell Cultures (ECACC), Japanese Collection of Research Bioresources (JCRB) cell bank, and Riken Bioresource Center. Cellosaurus is a knowledge resource that aims to describe all cell lines used in biomedical research by providing information on immortalized cell lines, including cancer cell lines [18]. There are 417 human pancreatic cancer cell lines, of which 377 are PDAC cell lines (Figure 1).

As of 20 April 2022, there are 417 pancreatic cancer cell lines, including 377 PDAC and 40 other pancreatic cancer cell types, in the Cellosaurus. In the PDAC cells, two types of cells mainly express epithelial or mesenchymal molecules. PANC-1 and MIA PaCa-2 have been classified into mesenchymal molecule-expressing PDAC cells and named quasi-mesenchymal or mesenchymal types in previous manuscripts [9,16]. The cell names in the figure are the names published in available papers. Colo357 is classified as adenosquamous carcinoma in the Cellosaurus. PDAC: Pancreatic Ductal Adenocarcinoma; PAC: Pancreatic Adenocarcinoma; PC: Pancreatic Carcinoma; PDC: Pancreatic Ductal Carcinoma; IPMN: Intraductal Papillary Mucinous Neoplasm; ITPN: Intraductal Tubulopapillary neoplasm; MCN: Mucinous Cystic Neoplasm; Classical and Quasi-Mesenchymal [9]; Epithelial and Mesenchymal [16]

## 3. Cancer Stem Cells in PDAC Cell Lines

According to the 2006 symposium of the American Association of Cancer Research, a CSC is defined as “a cell within a tumor that possesses the capacity to self-renew and cause the heterogeneous lineages of cancer cells that comprise the tumor” [19]. Three major methods are used to identify CSCs from various organs: CSC-specific marker detection, detection of side population (SP) cells, and sphere-forming assays [20]. These methods are used to identify CSCs in cultured cancer cell lines, while CSC-specific markers are used to identify CSCs in human cancer tissues. In pancreatic cancer, CSCs were first reported in 2007 as a subpopulation of CD44(+)/CD24(+)/ESA(+) or CD133(+)/CXCR4(+) cancer cells [21,22], and many CSCs have been identified using these three methods [23].

### 3.1. CSC-Specific Markers

CSC-specific markers are highly expressed in the cell membranes or cytoplasm of CSCs, and in human PDAC tissues, these markers can be detected using immunohistochemical and immunofluorescent staining. Pancreatic CSC markers including CD133, CD44, CD24, CXCR4, ATP-binding cassette sub-family G member 2 (ABCG2), ALDH1, nestin, and epithelial cell adhesion molecule (EpCAM, also known as ESA) are detected in PDAC tissues at various levels (13.5–78.9%) [20,24]. In human PDAC cell lines, CSC marker-positive cells were detected using flow cytometry. We previously reported that CD24 (0.071–45.3%), CD44 (46.1–100%), CD133 (0–1.61%), CXCR4 (0.274–38.2%), ESA (1.36–93.7%), and nestin (0.662–11.5%) were expressed in three human PDAC cell lines [24]. However, no unique marker has been identified for the isolation of CSCs from PDAC; therefore, a combination of several CSC markers such as CD133(+)/CXCR4(+), CD24(+)/CD44(+), CD24(+)/CD44(+)/ESA(+), c-Met(+)/CD44(+), and ALDH1(+)/CD133(+) might increase the purity of isolated CSCs [23,25]. CD44v6 and CD44v9, splice variants of CD44, were reported to be CSC markers for PDAC [26,27,28,29]. The regulatory mechanisms of CSC marker expression in PDAC cells are not well clarified. We have previously reported that nestin expression in PANC-1 cells is regulated by the methylation of the promoter region of the nestin gene [30].

### 3.2. Side Population Cells

Since CSCs more efficiently efflux anticancer drugs than non-CSCs, SP cells that rapidly efflux fluorescent dyes were selected as CSCs by flow cytometry. Zhou et al. showed that there were 2.1–8.7% of viable SP cells in PANC-1 cell populations [31]. SP cells exhibit an enhanced capacity for gemcitabine and Hoechst 33342 dye efflux; thus, PANC-1 cells have a significant survival advantage. We previously detected SP cells in PANC-1 cell populations from metastatic tumors in immunodeficient mice at approximately twice the proportion of that in counterpart parental PANC-1 populations [32]. The injection of the SP fraction from the KLM-1 PDAC cell line, which was established from the same patient as PK-1 cells, resulted in a larger tumor volume than an injection of the same number of cells from the major population (MP) [20]. These results indicate that SP cells contribute to the tumorigenesis and metastasis of PDAC.

### 3.3. Sphere-Forming Ability

The sphere formation assay indicates the self-renewal capacity of cancer cells that can form floating colonies called spheres when cultured in low-attachment dishes. PDAC cell lines can form spheres in low-attachment dishes, and sphere-forming cells possess stem cell abilities [33,34,35]. Furthermore, sphere-forming cells exhibited higher tumor formation rates than non-sphere-forming cells. CD44(+)/CD24(+) fractions from pancreatic tumors are enriched in sphere-forming cells [34], and nestin, which is a CSC marker of PDAC, had increased expression in the spheres of the three PDAC cell lines than in the non-sphere cells [30]. Compared with 2D culture conditions, CSC markers including ALDH1, Oct4, Nanog, CD24, and CD44v9 were highly expressed in the spheres of MIA PaCa-2 cells [28]. Oct4, Nanog, Sox2, CD24, and CD44v9 are more highly expressed in PANC-1 and PK-1 cells in spheres than in 2D culture conditions [29].

## 4. Morphology and Features of PDAC Cell Lines Grown in 2D Culture

**Figure 2:**
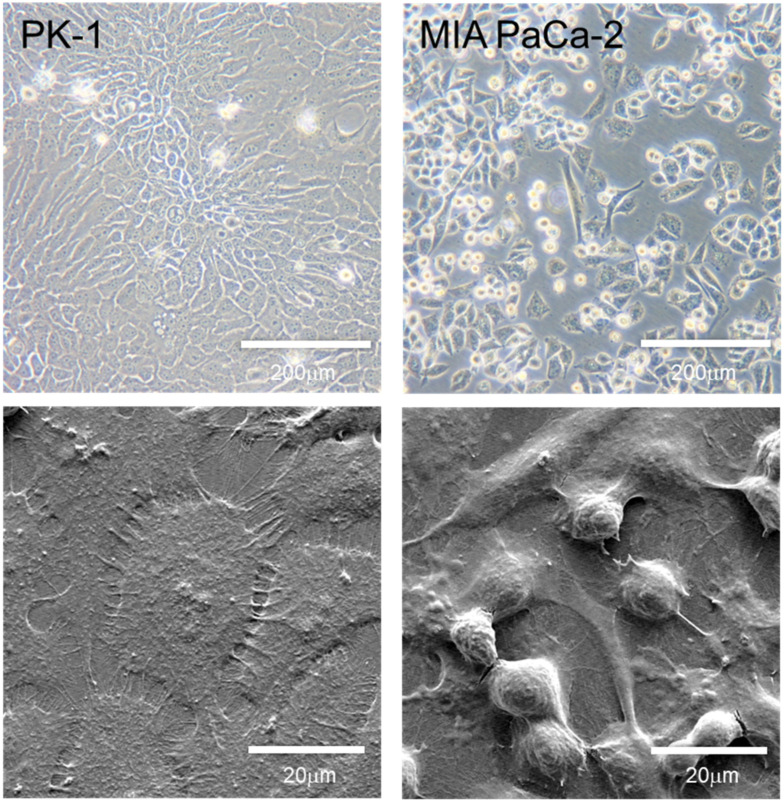
Phase-contrast and scanning electron microscopic images of PDAC cell lines grown in 2D culture conditions.

Although genetic abnormalities are different in each PDAC cell line, differences in their biological behavior have not been well investigated. Therefore, some medical journals require that more than two cancer cell lines show similar experimental results, and reviewers often point out that authors should perform the same experiments using more than one PDAC cell line. When cultured in 2D, most PDAC cells showed similar pleomorphic morphology (Figure 2, upper panels), but a small number of PDAC cell lines, including MIA PaCa-2, PK-59, and PK-45P cells, were mixed with pleomorphic- and spindle-shaped cancer cells [36]. PDAC cells can be classified into epithelial or quasi-mesenchymal phenotypes [37]. Transcriptional analyses of PDAC tissue samples and cell lines have suggested three molecular subtypes of PDAC: classical, quasi-mesenchymal, and exocrine-like subtypes [9]. The classical subtype is characterized by the expression of epithelial- and adhesion-related genes, whereas the quasi-mesenchymal subtype expresses mesenchymal-related genes. The exocrine-like subtype is characterized by the expression of genes associated with digestive enzymes. While the classical subtype showed the best prognosis, the quasi-mesenchymal subtype had the worst prognosis. In addition, the classical PDAC cell line subtype is resistant to gemcitabine but sensitive to erlotinib, while the quasi-mesenchymal subtype has the opposite resistance profile [9]. Molecular subtype classification was based on whole-exome sequencing and copy number variation analysis [38]. These results partially overlapped with the transcriptional analysis reported by Collison et al. [9]. Furthermore, molecular subtypes are associated with genomic stability and instability, and the genomic instability of the unstable subtype may be sensitive to DNA-damaging therapy [39]. Recently, the subtype classifications were brought into question because the exocrine-like and ADEX subtypes may have been due to contamination of normal acinar cells in the tumor tissues [40]. In addition, the Bailey’s squamous molecular subtype does not correspond to squamous differentiation at the histopathological level.

The PK-1 PDAC cells have a polymorphic morphology representative of most PDAC cell lines when cultured in 2D conditions [16,37]. However, the MIA PaCa-2 PDAC cells present with two types of cancer cell morphologies: polymorphic- and spindle-shaped cells [16]. These PDAC cells were cultured on regular culture plates and observed using phase contrast (upper panels) and scanning electron microscopy using secondary electrons (lower panels). (Document S1: Supplementary Materials and Methods).

In 2D culture conditions, we examined the epithelial or mesenchymal features of PDAC cell lines. Among the eight PDAC cell lines (PK-8, PK-45P, PK-59, PK-1, T3M-4, PANC-1, KP4 and MIA PaCa-2), five epithelial cell lines written earlier had high E-cadherin mRNA expression and low vimentin mRNA expression, whereas three mesenchymal cell lines written later expressed low E-cadherin and high vimentin expression [16,37] (Table S1). There was a 120,000-fold difference in the mRNA levels between PK-8 cells and MIA PaCa-2 cells, which had the highest and lowest E-cadherin expression, respectively [16]. The protein expression levels of E-cadherin and vimentin in PDAC cell lines corresponded to their respective mRNA expression levels. The complete methylation of E-cadherin promoters may correlate with the extremely low expression of E-cadherin in MIA PaCa-2 cells [41]. The expression of epithelial or mesenchymal-related proteins other than E-cadherin and vimentin in PDAC has been investigated in fibroblast growth factor receptors (FGFRs). Alternative splicing of the C-terminal half of the third immunoglobulin-like domain generates isoforms IIIb and IIIc in FGFR1-3 [42,43]. The IIIb isoform is mainly expressed in epithelial cells, while the IIIc isoform is expressed in mesenchymal cells [44]. PK-1 and PK-8 cells with epithelial features highly express the IIIb splicing isoforms in FGFR1 and FGFR2 [45]. In contrast, the IIIc mesenchymal isoforms in FGFR1 and FGFR2 were highly expressed in PDAC-1 and MIA PaCa-2 cells, which are PDAC cells with mesenchymal features. The overexpression of FGFR2IIIc isoforms increases the proliferation, migration, and invasion abilities of cultured PDAC cell lines [45]. In addition, PDAC cells that overexpress FGFR2 IIIc formed larger primary tumors and more liver metastases in severe immunodeficient NOD/Shi-*scid*/IL-2Rγ^null^ (NOG) mice after implantation of the cells into the pancreas [45]. Epithelial splicing regulatory protein 1 (ESRP1) is an RNA-binding protein that helps enhance splicing of the upstream exon IIIb and silence the downstream exon IIIc binding intronic splicing enhancer/intronic splicing silencer-3 (ISE/ISS-3). ESRP1 induces FGFR2 IIIb isoform expression and suppresses IIIc isoform expression. ESRP1-overexpressing PDAC cells had high FGFR2 IIIb levels and low migration and invasion abilities [46]. These PDAC cells also formed significantly fewer liver metastases than the control cells in NOG mice. ESRP1 also modulates the splicing of additional mRNA moieties in PDAC cells, including *FGFR1*, *FGFR3*, and *CD44* [46]. It is still unclear why there are different epithelial and mesenchymal features in PDAC cell lines and whether there are mutual changes that contribute to the epithelial and mesenchymal features. PK-1 cells possess SMAD4 genetic abnormalities and are not immunostained with an anti-SMAD4 antibody [37]. TGF-β1 administration did not induce an epithelial–mesenchymal transition in PK-1 cells, which retained epithelial-like features. Further studies are needed to clarify how epithelial and mesenchymal features are acquired and how they affect the cellular behavior of PDAC cell lines.

## 5. Morphology of Epithelial and Mesenchymal Features in PDAC Spheres Grown in the 3D Culture

**Figure 3:**
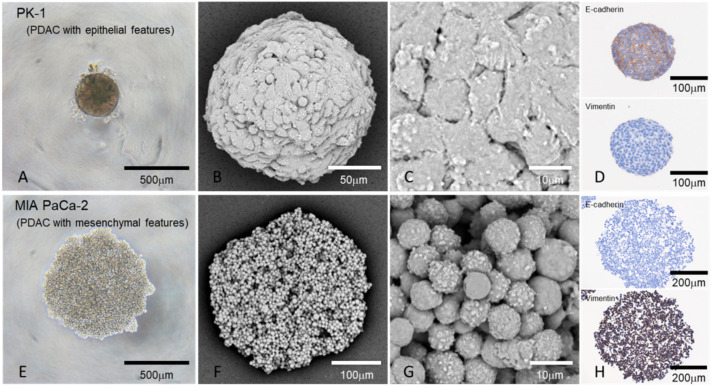
Morphology and epithelial or mesenchymal features of PDAC cell lines grown in 3D culture. PK-1 cells that have epithelial features formed small, firm spheres with slightly larger cancer cells that adhered to each other at the surface of the spheres (**A**–**C**) [16,37]. In contrast, MIA PaCa-2 cells with mesenchymal features formed large and loose spheres without fusion of the cancer cells (**E**–**G**) [16]. The PK-1 spheres were strongly positive for E-cadherin and negative for vimentin by immunostaining (**D**), while MIA PaCa-2 showed the opposite staining pattern for these markers (**H**). PK-1 and MIA PaCa-2 cells were cultured in low attachment plates with 10% fetal bovine serum containing RPMI1640 medium for 7 days. Left panels (**A**,**E**): phase-contrast images; middle panels (**B**,**C**,**F**,**G**): scanning electron microscopy with reflected electrons; right panels (**D**,**H**): immunocytochemical analyses using cell blocks of spheres. (Supplementary Materials and Methods).

Pathologically, pancreatic cancer cells proliferate sterically in both primary and metastatic lesions, forming masses in the human body. By culturing PDAC cells on a low-attachment plate using the same medium as the 2D culture, 3D spheres can be formed [35]. The expression levels of most CSC-specific markers in these spheres were higher than those in adherent cultures of the same cell lines, suggesting that CSCs are abundant in these spheres [29]. Significant differences in the morphology of PDAC cells grown in 3D cultures compared with 2D cultures have been reported [16,29,37]. PDAC cell lines with epithelial features (high E-cadherin and low vimentin expression) formed small spheres, whereas PDAC cells with mesenchymal features (low E-cadherin and high vimentin expression) produced large spheres. The surfaces of the spheres formed by PDAC cell lines with epithelial features were fused and formed surface-lining cells, while the surfaces of the spheres formed from PDAC cell lines with mesenchymal features were individually separated (Figure 3). Immunohistochemically, E-cadherin was strongly localized in the spheres formed by PDAC cells with epithelial features, whereas vimentin was not. In contrast, vimentin was strongly localized in spheres with mesenchymal features, while E-cadherin was not. The staining patterns of E-cadherin and vimentin in PDAC cells were stronger in 3D cultures than in 2D cultures.

## 6. Different Biological Behaviors of PDAC Cell Lines in 2D and 3D Culture Systems

Because PDAC forms 3D tumors in vivo even in the early stages, 3D culture is considered to be an important in vitro experimental condition. However, studies using PDAC cell lines are primarily based on the results of 2D culture systems. Previous studies have shown that there are differences in the proliferative capacity of cells and the effects of anticancer drugs on PDAC cells, as well as mucus secretion that is characteristic of adenocarcinoma in cells lines cultured in 2D and 3D conditions [16,17].

### 6.1. Cell Proliferation

Previous studies have reported that the proliferative capacity of cancer cells is higher in 2D cultures than in 3D cultures [47,48]. In most PDAC cell lines, adherent cells were more positive for the cell proliferation marker Ki-67 in 2D cultures than in 3D cultures [16]. Among the eight PDAC cell lines, seven showed a higher proliferative capacity in 2D culture than in 3D culture; Ki-67-positive PDAC cells were diffusely observed in 2D culture, while in 3D culture, Ki-67-positive cells localized in the periphery of the PK-1 cell spheres and gathered around the spheres of KP4 cells [16]. In some PDAC cell cultures, the cells around the sphere grow like a proliferation zone and expand toward the periphery. This in vitro evidence suggests that there are both expanding and infiltrating proliferation patterns to PDAC cells grown in vivo that depend on the cell type.

### 6.2. Anticancer Drug Resistance

Almost all anticancer drugs are less effective in multicellular spheroid models than in 2D cell cultures [47,49,50]. It has been reported that the 50% inhibitory concentration (IC_50_) for gemcitabine and oxaliplatin in PDAC cells needs to be 200-fold higher in 3D culture spheres than in 2D culture [51]. When the effect of the anticancer drug on the spheres in human PDAC cell line cultures was examined with an ATP assay, it was found that 100 µM gemcitabine was more effective against PDAC cell lines with epithelial features than mesenchymal PDAC cells, while 100 µM nab-paclitaxel was effective against PDAC cells with mesenchymal features [16]. A previous study showed that the mRNA levels of the multidrug resistant transporter ABCG2 in PANC-1 cells was approximately 2-fold higher in 3D-culture than in 2D-culture [52]. Further, the expression levels of four transporters (ABCG2, ABCB1, ABCC1, and ABCC2) that are involved in drug excretion in human PDAC cell line spheres were high in the PDAC cell lines that had mesenchymal features [16].

It is unclear why the effects of anticancer drugs differ between epithelial and mesenchymal PDAC cell lines grown in 3D culture conditions, but these features may contribute to the efficacy of anticancer drugs. The spheres of PDAC cells with epithelial features are covered with surface lining cells, while the spheres of mesenchymal-featured PDAC are uncovered and remain separated from one another. This difference may assist in anticancer drug permeability into the spheres. In addition, the spheres of PDAC cell lines with mesenchymal features have more proliferative activity than the spheres of epithelial PDAC cell lines, with the proliferating cells confined to the surface cells of the spheres in PK-1 [16], which may be linked to the high expression of drug excretion pumps in PDAC cell lines with mesenchymal features. Based on these findings, research on anticancer drug resistance using PDAC cell lines in 3D culture may contribute to the discovery of anticancer drugs that are more suitable for patient care.

## 7. Epithelial and Mesenchymal Features of Human PDAC Tissues

There have been several studies of the relationship between pancreatic cancer with low E-cadherin or vimentin expression and poor prognosis in human patients with PDAC [41,53]. Tissue microarray analysis conducted at Johns Hopkins University showed that 43% of PDAC cases had partial or complete loss of E-cadherin expression [53]. PDAC cases that do not express E-cadherin often have cancer cells that do not adhere to each other, which correlates with an extremely poor prognosis [41]. In autopsies of PDAC cases, E-cadherin-positive cancer cells accounted for 50% of the primary tumors, while the proportion of E-cadherin-positive cells is significantly lower in the liver and other metastatic lesions [32]. Histologically, a loss of E-cadherin is more commonly observed in poorly differentiated PDAC [54,55,56,57] while in contrast, 45% of PDAC cases had vimentin expressed in 1–95% of cancer cells, and 27.5% had vimentin expressed in 10% or more of the cancer cells [58]. Vimentin expression is an independent factor in the short postoperative prognosis of PDAC, which indicates that, similar to PDAC cell lines, human PDAC cases contain epithelial or mesenchymal cancer cell types. Therefore, the results of the 3D culture of PDAC cell lines may be applied to human PDAC tissues. Comparing immunostaining of PDAC cancer tissues and cancer cell lines established from the same patients may be effective in clarifying the relationship between cancer tissues and 2D or 3D cultured cell lines.

## 8. Future Perspective

There is abundant desmoplastic stroma around PDAC cells in human PDAC tissues. PDAC stroma consists of stromal and inflammatory cells, and extracellular matrix proteins that all contribute to the aggressive biological behavior of the tumor [59]. The tumor stroma of PDAC tissues is mainly created by activated pancreatic stellate cells (PSCs) [60]. In early-stage PDAC cases, moderate to strong smooth muscle actin expression in PSCs was associated with poorer clinical outcomes than those with low expression levels of α-smooth muscle actin [61]. Molecular subtyping of PDAC stroma identified “normal” and “activated” PDAC stroma subtypes, with the “activated” subtype being associated with a worse prognosis [62]. In addition to the heterogeneity of PDAC cells, research on the stroma surrounding PDAC cells is required for improved understanding and treatment of patient disease conditions. In the future, it will be necessary to develop a 3D co-culture method for PDAC cells with stellate cells, inflammatory cells, including macrophages and vascular endothelial cells to mimic PDAC tissue morphologies and features observed in the human body [63,64].

## 9. Conclusions

Although their morphology is almost unchanged when cultured in 2D conditions, PDAC cell lines established from patients with PDAC are divided into those that express epithelial or mesenchymal proteins. However, in 3D culture, epithelial PDAC cells form small spheres with coated cells on the surface, while mesenchymal PDAC cells form spheres that are loosely bound together. Epithelial and mesenchymal PDAC spheres differ in cell proliferation and anticancer drug responses in 3D culture. More selective in vitro research on the characteristics of PDAC and effective anticancer agents will be advanced by 3D co-culture methods of epithelial or mesenchymal PDAC cells and stromal cells present around the tumor.

## Data Availability

The datasets used and/or analyzed during the current study are available from the corresponding author upon reasonable request.

## Supplementary Materials

The following are available online at https://www.mdpi.com/article/10.3390/jpm12050746/s1, Table S1: Primer list, Document S1: Supplementary materials and methods.
