# Supplementary material for: Epithelial and Mesenchymal Features of Pancreatic Ductal Adenocarcinoma Cell Lines in Two- and Three-Dimensional Cultures
Source: J Pers Med. 2022 May 4;12(5):746. doi: 10.3390/jpm12050746 (PMC9146102; doi:10.3390/jpm12050746)
Supplement: Supplementary file 1 [file jpm-12-00746-s001.zip › jpm-1653022-supplementary.pdf]

Epithelial and Mesenchymal Features of Pancreatic Ductal Adenocarcinoma Cell Lines in 2- and 3-Dimensional Cultures

Yuuki Shichi[1], Fujiya Gomi[1], Norihiko Sasaki[2], Keisuke Nonaka[1], Tomio Arai[3], Toshiyuki Ishiwata[1*]

Document S1: Supplementary materials and methods

Search criteria for human pancreatic cancer cell lines in the Cellosaurus

A search for "pancreatic cancer" in the Cellsaurus revealed 629 cell lines, and narrowing down the species to *Homo sapiens* resulted in 527 cell lines. Data on cancers such as invasive breast carcinoma of no special type, breast ductal carcinoma, ductal cell of pancreas, high-grade ovarian serous adenocarcinoma, esophageal squamous cell carcinoma, and stellate cell were mixed; therefore, they were excluded (417 cell lines). The data obtained were then segregated to PDAC (377 cell lines) and other pancreatic cancer (40 cell lines) cell lines.

Cell culture

Human PDAC cell line PK-1 and MIA PaCa-2 cells were obtained from the Cell Resource Center for Biomedical Research, Institute of Development, Aging and Cancer, Tohoku University (Sendai, Japan). Cells were grown in a growth medium (RPMI-1640 medium containing 10% fetal bovine serum) at 37 °C in a humidified 5% CO_2 atmosphere. To form spheres, cells (3×10^3 cells/well) were plated in 96-well ultra-low attachment plates (Thermo Fisher Scientific, Waltham, MA, USA) with a growth medium. After 7 days, the spheres were photographed using a phase-contrast microscope (Eclipse TS-100, Nikon, Tokyo, Japan). Spheres were then aspirated using micropipettes and used for further experiments.

Scanning electron microscopy (SEM)

Adherent PK-1 and MIA PaCa-2 cells were fixed for 30min, and PK-1 and MIA PaCa-2 spheres were fixed overnight with 2.5% glutaraldehyde in 0.1 M phosphate buffer (pH 7.4) at 4°C. Then, the glutaraldehyde solution was removed, and the cells were washed with phosphate-buffered saline. The PDAC cells were post-fixed with osmium tetroxide for 30 min. After complete dehydration via a graded ethanol series, samples were suspended in 100% ethanol, air-dried, and coated with a platinum layer using an MSP-1S sputter coater (Shinku Device, Ibaraki,

Japan). Cells were photographed with a Phenom Pro desktop scanning electron microscope using secondary electrons and reflected electrons for attached cells and spheres, respectively (Thermo Fisher Scientific).

Immunocytochemical analysis of cell blocks for spheres

The spheres were collected using a micropipette under a microscope and then fixed in 10% neutral-buffered formalin for 3 h. Formalin was removed using a micropipette, and embedded in paraffin. Serial sections of the cell blocks (4-μm thickness) were immunostained using the labeled streptavidin-biotin method. The primary antibodies used in immunocytochemical staining were as follows: mouse monoclonal anti-E-cadherin antibody (M106) from Takara Bio (Shiga, Japan) and mouse monoclonal anti-vimentin antibody (422101) from Nichirei Biosciences Inc. (Tokyo, Japan). Sections were treated with 0.03% H_2O_2 in 33% methanol at room temperature for 30 min to block endogenous peroxidase before antigen retrieval treatment. The reaction to each antigen was visualized by adding 3,3′-diaminobenzidine tetrahydrochloride (DAB) and counterstaining with hematoxylin. The images were taken with an upright microscope (BX43; Olympus, Tokyo, Japan).

Table S1. Primer list [15, 34]

Gene	Forward primer	Reverse primer
E-cadherin	CCAGTGAACAACGATGGCATT	TGCTGCTTGGCCTCAAAAT
Vimentin	TCCAAACTTTTCCTCCCTGAAC	GGGTATCAACCAGAGGGAGTGA
β-ACTIN	GGTCATCACCATTGGCAATGAG	TACAGGTCTTTGCGGATGTCC
